# The therapeutic prospects of N-acetylgalactosamine-siRNA conjugates

**DOI:** 10.3389/fphar.2022.1090237

**Published:** 2022-12-14

**Authors:** Lei Zhang, Yayu Liang, Guohui Liang, Zhili Tian, Yue Zhang, Zhihui Liu, Xinying Ji

**Affiliations:** ^1^ Henan International Joint Laboratory of Nuclear Protein Regulation, School of Basic Medical Sciences, Henan University, Kaifeng, China; ^2^ School of Stomatology, Henan University, Kaifeng, China; ^3^ School of Clinical Medical Sciences, Henan University, Kaifeng, China; ^4^ Department of Obstetrics and Gynecology, Zhengzhou, China; ^5^ Department of General Practice, Henan Provincial People’s Hospital, Zhengzhou University, Zhengzhou, China

**Keywords:** RNAi, GalNAc-siRNA, delivery system, ASGPR, LNPs

## Abstract

RNA interference has become increasingly used for genetic therapy following the rapid development of oligonucleotide drugs. Significant progress has been made in its delivery system and implementation in the treatment of target organs. After a brief introduction of RNA interference technology and siRNA, the efficiency and stability of GalNAc-siRNA conjugates are highlighted since several oligonucleotide drugs of GalNAc have been approved for clinical use in recent years. The structure and features of GalNAc-siRNA conjugates are studied and the clinical efficiency and limitations of oligonucleotide-based drugs are summarized and investigated. Furthermore, another delivery system, lipid nanoparticles, that confer many advantages, is concluded, includ-ing stability and mass production, compared with GalNAc-siRNA conjugates. Importantly, developing new approaches for the use of oligonucleotide drugs brings hope to genetic therapy.

## 1 Introduction

RNA interference (RNAi) is a natural defense mechanism widely present in organisms to protect against exogenous gene invasion (Svoboda, 2014; Swevers et al., 2018). It is one of the most important methods, together with zinc-finger nucleases, transcription activator-like effector nucleases and clustered regularly interspaced short palindromic repeat/associated protein, for regulating gene expression and growth and offers significant application value for the research of gene function and the development of gene therapeutics (Hannon, 2002; Xu et al., 2022). Specifically, RNAi primarily downregulates target gene expression *via* sequence specificity involving specific enzymatic degradation of target mRNA by the RNA-induced silencing complex (RISC) mediated by the siRNA antisense chain (Han, 2018). In other words, siRNA is a highly specific biomolecule that can inhibit or silence the expression of its complementary genes. In addition, this inhibition or silencing effect (degradation reaction) has a cascade effect (Liu et al., 2017). The benefits of RNA interference technology include high efficiency, targeting and low toxicity, accounting for its widespread use in drug research (Saw and Song, 2019; Setten et al., 2019). In August 2018, the US Food and Drug Administration and the European Commission approved ONPATTRO (Patisiran), which is the first approved for clinical use, developed by Alnylam Pharmaceuticals, to treat peripheral neuropathy with hereditary transthyroxine protein-mediated (hATTR) amyloidosis (Hoy, 2018). This RNAi milestone for the pharmaceutical industry represents one of the breakthroughs of oligonucleotide drug development, from research to clinical application.

N-acetylgalactosamine (GalNAc) conjugated to siRNA is considered as a promising solution of siRNA delivery system. The GalNAc GalNAc is targeting ligand binds highly selectively to asialoglycoprotein (ASGPR), which is abundantly expressed on hepatocyte cell, resulting in rapid endocytosis (Nair et al., 2014; Crooke et al., 2018; Jeon et al., 2022). The formed GalNAc-siRNA conjugates target ASGPR specifically expressed on the surface of hepatocytes, assisting in cleaving and separating approved durgs including Leqvio^®^ (inclisiran), GIVLAARI™ (givosiran), Oxlumo™ (lumasiran), and AMVUTTRA™ (vutrisiran) as targeted RNA strands. These RNA strands can silence mRNA to lowering abnormal metabolites level and eventually ease the symptoms and pain (Bissell et al., 2017; German and Shapiro, 2020; Zhang et al., 2021a; Cui et al., 2021; Dobrowolski et al., 2021; Scott and Keam, 2021; Storjord, 2021; Yu and Tu, 2021; Keam, 2022).

In this manuscript, several critical features are included. The structure and mechanism of action are priorly mentioned, following by the chemical modification, clinical and preclinical advances as well as challenges and limitations of GalNAc-siRNA conjugates. Finally, the features of GalNAc-siRNA conjugates are concluded by comparing GalNAc-siRNA conjugates to lipid nanoparticles (LNPs) to highlight the safety and efficiency of GalNAc-siRNA conjugates.

## 2 Challenge and siRNA delivery system

Despite its potential advantages, siRNA is beset with difficulties in clinical application (Xu and Anchordoquy, 2011; Khan, 2019). The main difficulty that restricts the implementation of siRNAs into clinical practice is drug delivery, given the poor molecular stability, wide distribution *in vivo*, difficulty in cell uptake, high dose, poor targeting, and wide variety of biological barriers, such as the GI tract mucosal epithelium, nasal/lung epithelia and skin, which affect oral, nose/pulmonary and dermal/transdermal administration, respectively (Antimisiaris et al., 2021). Moreover, naked siRNA can be easily degraded by RNases, thus failing to perform targeted knockdown (Springer and Dowdy, 2018). Additionally, the negatively charged hydrophilic phosphate groups in the double-stranded skeleton structure of siRNA make it difficult for the cell membrane to absorb naked siRNA. Therefore, siRNA needs to be chemically modified or delivered with the help of vectors. In the meantime, exogenous siRNA may compete with endogenous RNA, resulting in supersaturation. Exogenous siRNA also causes an “off-target effect”, leading to the silenced expression of nontarget functional genes, significant siRNA side effects, and intracellular trafficking, which is a significant contributing factor to oligonucleotide delivery (Juliano, 2018; Marinho et al., 2018). Importantly, the above problems can be minimized or avoided by designing reasonable, efficient and specific siRNA sequences or chemical modifications (such as pentose modification and base modification). Oligonucleotide drugs have achieved sustained significant progress in treating various diseases in the recent decade. As therapeutics that selectively suppress target genes through the mechanism of RNA interference, oligonucleotide drugs have been granted market approval (Cui et al., 2021). For instance, Leqvio^®^ (inclisiran), GIVLAARI™ (givosiran), Oxlumo™ (lumasiran), and AMVUTTRA™ (vutrisiran) that are approved for clinical usage, can target liver mRNAs for the treatment of hypercholesterolemia, mixed dyslipidemia, acute hepatic porphyria (AHP), primary hyperoxaluria type 1 (PH1) and hATTR amyloidosis (Lamb, 2021; Scott and Keam, 2021; Subhan et al., 2021; Adams et al., 2022). Importantly, more oligonucleotide drugs that target metabolic dysfunction symptoms and subsequent complications are under development, providing hope for more genetic solutions.

Common nonviral vectors based on RNAi include LNPs, cationic polymers that represent polymer-based delivery systems and siRNA-carbohydrate bioconjugates such as GalNAc-siRNA conjugates (Subhan et al., 2021; Tian et al., 2021; Thapa Magar et al., 2022). At present, GalNAc-siRNA conjugates and LNPs are the most studied and discussed delivery systems in RNAi therapy owing to their practicality, stability, and safety (Paunovska et al., 2022).

## 3 GalNAc-siRNA Conjugates

Twenty years after RNAi discovery, siRNA therapy has begun to be implemented in clinical practice. In recent years, unprecedented progress has been achieved in developing various delivery technologies. One giant leap in delivery technology is the GalNAc-siRNA conjugate, which specifically delivers to and targets the liver. In November 2019, GIVLAARI^®^ (givosiran), as the first GalNAc siRNA drug, was approved to be marketed in the United States for the treatment of acute hepatic porphyria, followed by inclisiran and lumasiran (Table 1) (Scott, 2020). The nature of the chemical mechanism of these approved drugs is the binding of siRNA to GalNAc.

**TABLE 1:** Marketed GalNAc-conjugated RNA drugs.

Brand name	Generic name	Target	Molecular mechanism	Condition
Leqvio	Inclisiran	proprotein convertase subtilisin/kexin type 9 (PCSK9)	PCSK9 Expression Inhibitors	atherosclerosis, dyslipidemia, hypercholesterolemia, familial hypercholesterolemia, hyperlipidemia
Oxlumo	Lumasiran	hydroxyacid oxidase 1 (HAO1)	HAO1 Expression Inhibitors	end-stage renal disease, hyperoxaluria, primary hyperoxaluria type 1
Givlaari	Givosiran	5′-aminolevulinate synthase 1 (ALAS1)	ALAS1 Expression Inhibitors	porphyria, acute porphyria
Amvuttra	Vutrisiran	transthyretin (TTR)	TTR (Mutant) Expression Inhibitors	amyloidosis, transthyretin-related amyloidosis

### 3.1 Structure and mechanism of action

**FIGURE 1:**
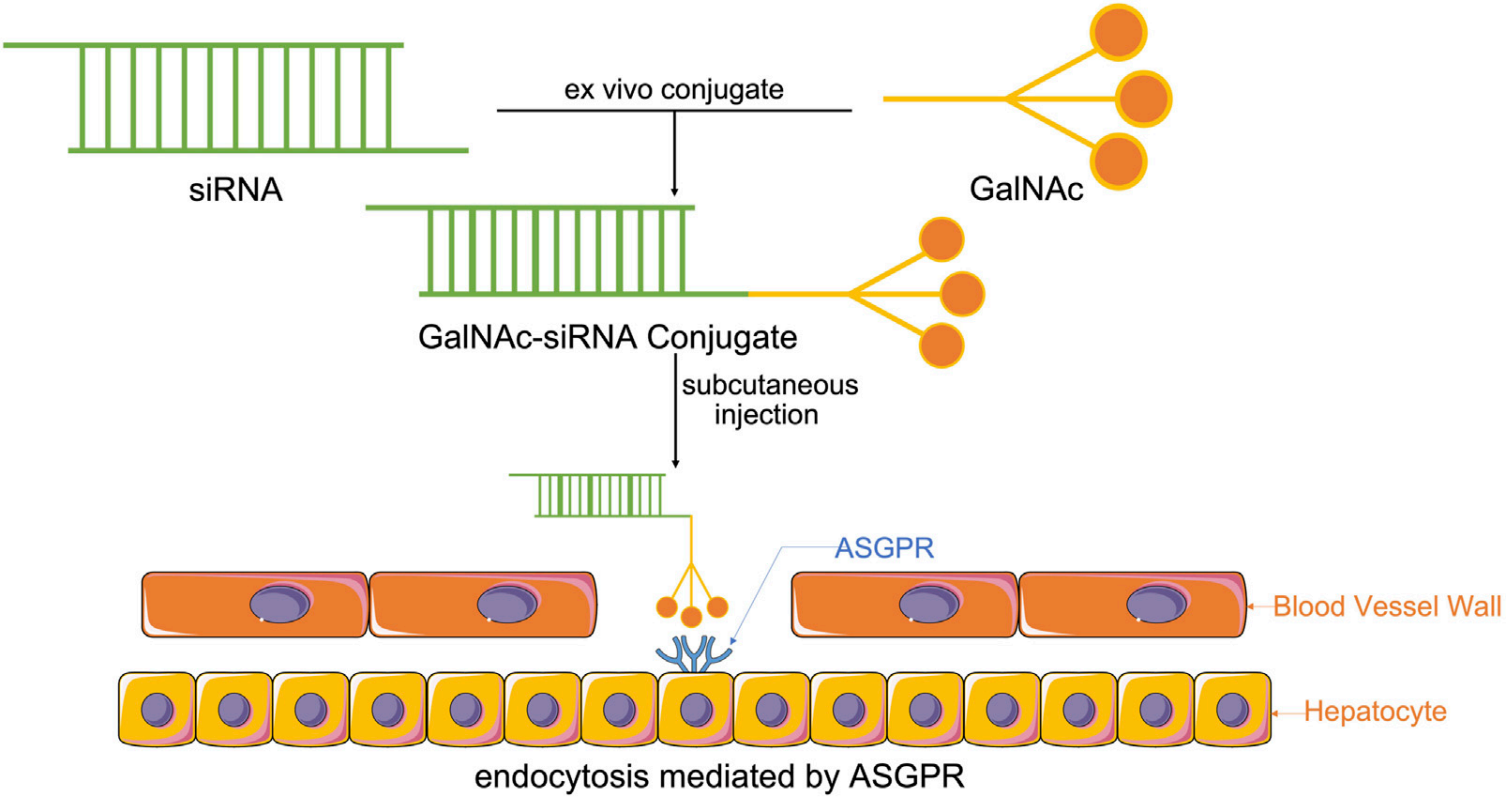
Synthesis of GalNAc-siRNA conjugates. Through chemically synthesized conjugates, modified siRNAs with GalNAc enter the system by subcutaneous injection, which is the most effective way to help GalNAc-siRNA conjugates attach to ASGPR, the receptor, that is, chiefly expressed on the surface of liver parenchyma cells in the hepatic sinusoid space, thus enabling ASGPR-mediated cellular endocytosis to cause siRNA to enter the cell and perform its function.

The coupling of drugs with cell surface receptor ligands has been reported as a promising pathway for targeted drug delivery. These receptors are only expressed in particular cell types and are overexpressed in specific organs or tissues in certain diseases. GalNAc is an efficient nucleic acid therapeutic ligand with a high affinity for ASGPR. ASGPR, also called the Ashwell-Morell receptor, has been established to feature liver specificity and species specificity. It is chiefly expressed on the surface of liver parenchyma cells in the hepatic sinusoid space and exhibits calcium-dependent ligand binding (Gabba et al., 2021). Due to the evolution of human defense mechanisms, negatively charged siRNAs with large molecular weights cannot enter the cell itself. Accordingly, siRNAs with GalNAc have been modified for targeted siRNA delivery to the liver and siRNA entry into the cell *via* ASGPR-mediated cellular endocytosis (Figure 1) (Ayyar et al., 2021; Zhou et al., 2021; Paunovska et al., 2022).

**FIGURE 2:**
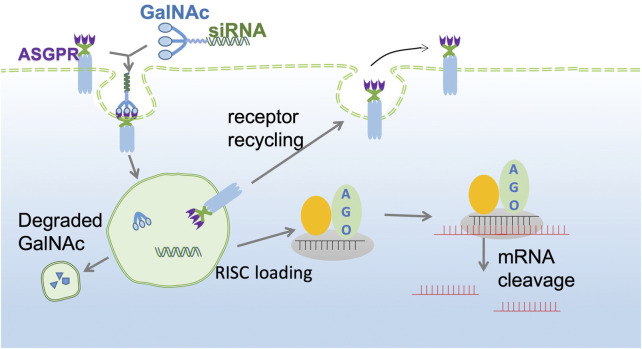
GalNAc-siRNA conjugate pathway. The GalNAc-siRNA conjugate binds to ASGPR receptors on the surface of hepatocytes and is then endocytosed into the cytoplasm to form endosomes. Due to the decrease in pH in endosomes, the siRNA GalNAc-ASGPR complex is decomposed, and less than 1% of free siRNA escapes to the cytoplasm to exert an RNAi effect. ASGPR will return to the surface of liver cells for recycling, while GalNAc will be degraded and excluded. The negative chain of siRNA, Ago2 protein and related enzymes together constitute RISC and guide RISC to bind to target mRNA to achieve target mRNA gene silencing.

The GalNAc trimer is mainly synthesized by D-(+)-galactosamine (I), 2-amino-2-hydroxymethyl-1,3-propanediol (II), and trans-4-hydroxy-L-proline methyl ester (III). Solid carrier IV plus tyrosine can be used as a radioactive marker to evaluate the *in vivo* receptor-ligand interactions and GalNAc-siRNA conjugate uptake (Nair et al., 2014). Subsequently, the synthesized three-antenna GalNAc can combine with the 3′ end of the antisense chain of siRNA to form a GalNAc-siRNA conjugate (Shchegravina et al., 2021). siRNA can be synthesized directly by a chemical approach or by breaking long double-stranded RNA obtained by enzyme transcription into 21–23 nt siRNA under the action of the cytoplasmic RNase III endonuclease Dicer, which has two protruding nucleotides at the 3′ hydroxyl end, a phosphate terminal at the 5′ end and a complementary double-stranded region of 19 nt in the middle (Adachi et al., 2021; Paro et al., 2021). When GalNAc-siRNA is introduced into the body and binds to ASGPR on the surface of hepatocytes, the siRNA GalNAc-ASGPR complex is separated in the endosome (Holland et al., 2021; Shchegravina et al., 2021). Thereafter, siRNA escapes from the endosome, and GalNAc is degraded and excreted out of the endosome. Vesicles enclosing the ASGPR are fused with the cell membrane to return to the surface of hepatocytes to complete the ASGPR cycle (van den Berg et al., 2021; Damase et al., 2021). After the escaped or released siRNA molecules separate the double strand under the action of helicase, the negative strand binds to Ago2 proteins and related enzymes to form RISC and guide RISC to bind to the complementary regions of the target dsRNA. Under the action of Ago2 protein, the newly combined double-stranded complex can break the phosphodiester bond between the 10th and 11th bases at the 5′ end of antisense RNA to achieve siRNA-mediated gene silencing of specific target mRNAs (Figure 2) (Alshaer et al., 2021; Iwakawa and Tomari, 2021; Montañés et al., 2021; Zhao et al., 2021).

### 3.2 Chemical modification

**FIGURE 3:**
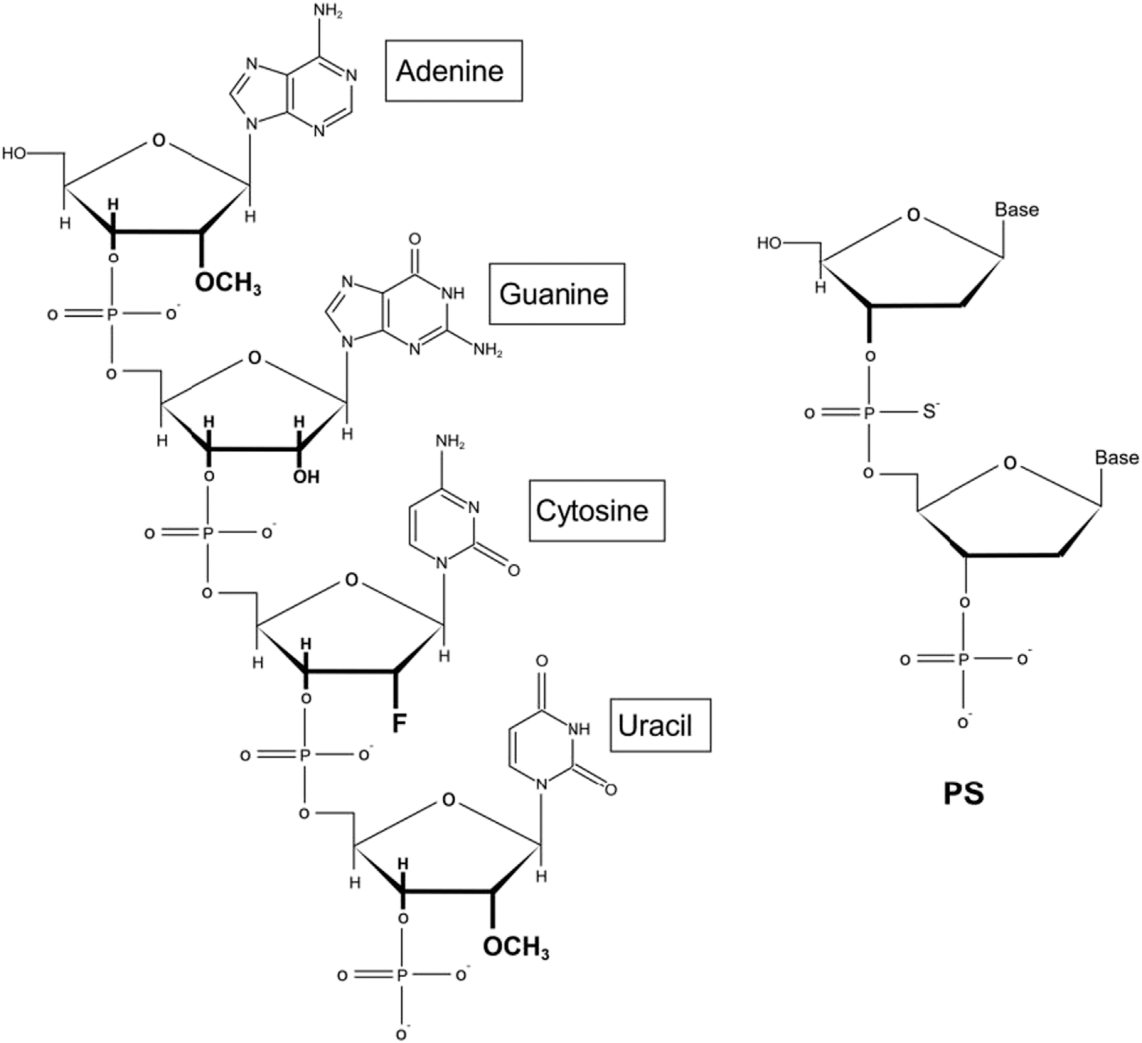
Schemes of siRNA chemical modification.

Moreover, the chemical modification of siRNA is conducive to further application of siRNA in the clinic. Initially, the experimenters try to modify the phosphate skeleton, base, terminal groups and ribose according to the structure of siRNA. The results show that ribose modification is more advantageous to some extent. Among them, the chemical modification of ribose 2′-OH is the most important. It is widely acknowledged that oligonucleotide nuclease stability can be significantly refined by adjusting the 2′ position of the RNA. In addition, it has been shown that by further improving the siRNA chemically, such as through optimizing the position of ribose modification of 2′-deoxy-2′–fluoro (2′-F) and 2′-O-methyl (2′-OMe) on the two strands of double-stranded siRNA (Figure 3), the stability can be improved without affecting inherent RNAi activity to achieve substantial therapeutic improvement (Foster et al., 2018). This phenomenon has also led to the development of standard template chemistry (STC) toward enhanced stabilization chemistry (ESC) and advanced ESC. Early GalNAc-siRNA conjugates (STC) modified with 2′-F or 2′-OMe are stable enough to stimulate activity *in vivo* but require high doses. ESC is designed to add two phosphorothioate (PS) bonds at the 5′ ends of the siRNA guide chain and the guest chain (Foster et al., 2018; Brown et al., 2020). An increasing body of evidence suggests that ESC siRNA exhibits enhanced effectiveness and duration in preclinical and clinical trials. However, the good tolerance of siRNA containing 2′-F and 2′-OMe modifications has led to advanced ESC designs, which achieve optimal results by adjusting the position and ratio of 2′-F and 2′-OMe in the double strand (Nair et al., 2017; Gupta et al., 2021a). Although both designs yield steady results during *ex vivo* studies, it is worth noting that compared with *in vivo* ESC templates, the efficacy and duration of advanced ESC designs are superior (Brown et al., 2020; Kulkarni et al., 2021). In conclusion, the above results demonstrate that the covalent bonding of the GalNAc trimer with optimal, chemically modified siRNAs yields a conjugate with nuclease stability and improves the pharmacokinetics compared to uncoupled siRNAs, resulting in a steady increase in the potency and duration of GalNAc-siRNA conjugate activity (Lundin et al., 2015; Freitag and Wagner, 2021).

Furthermore, nonclinical studies on ultratherapeutic doses of GalNAc-siRNA have shown that some typical molecules (six enhanced stable chemical GalNAc-siRNAs) exhibit similar safety signals and histological findings in the liver, as well as the kidney and lymph nodes (Janas et al., 2018a; Sutherland et al., 2020). Most of these conjugates have no side effects, which can be attributed to their pharmacokinetics and intracellular distribution, which usually reflect the cumulative dose efficiency (Debacker et al., 2020). In addition, all GalNAc-siRNAs assessed thus far have been shown to be nongenotoxic and have undergone pharmacological safety studies (Janas et al., 2018a).

### 3.3 Clinical and preclinical advances in approved or ongoing GalNAc-siRNA therapeutics

To date, three GalNAc-siRNA therapeutics, Leqvio^®^ (inclisiran), GIVLAARI™ (givosiran) and Oxlumo™ (lumasiran), have been approved for commercial applications, with 13 GalNAc-siRNA products undergoing clinical trials (Supplementary Table S1). Abundant preclinical studies are implementing or awaiting further improvements (Supplementary Table S2). The clinical advances of GalNAc conjugates for treating AHP, hypercholesterolemia, PH1, etc. are concluded.

#### 3.3.1 Clinical efficacy of several approved GalNAc-siRNA conjugates drugs: Leqvio^®^ (inclisiran), GIVLAARI™ (givosiran), Oxlumo™ (lumasiran), and AMVUTTRA™ (vutrisiran)

As previously mentioned, GalNAc-siRNA conjugates are commonly used in liver diseases (Springer and Dowdy, 2018; Willoughby et al., 2018; Thangamani et al., 2021). The development of GalNAc-siRNA conjugates can help to boost oligonucleotide drug popularity by removing barriers such as poor drug safety, efficacy, and specificity posed by other delivery systems. Promptly internalized by high-capacity ASGPR, GalNAc conjugates can internalize within clathrin-coated vesicles and enter endosomal compartments, leading to the disruption of ionic interactions caused by endosomal acidification. In this regard, decreasing the pH of endosomal compartments can help to release GalNAc-siRNAs from ASGPRs to transfer GalNAc-siRNAs back to the cell surface every 10–15 min, ensuring transport efficacy (Brown et al., 2020; Abdelaal and Kasinski, 2021). However, it has been shown that only a few (<1%) GalNAc-siRNA conjugates are distributed from pH-decreasing endosomal compartments to the cytoplasm, making endosomal escape an important rate-determining step for the efficient delivery of siRNAs and antisense oligonucleotides (ASOs) (Gilleron et al., 2013; Springer and Dowdy, 2018; Ayyar et al., 2021; Holland et al., 2021). Potential endosomal-escape promoters such as chloroquine and nigericin, also known as osmotic agents, along with other approaches and techniques, have been associated with cytotoxicity that has limited their therapeutic application, making GalNAc-siRNA conjugates more promising solutions (Lechanteur et al., 2018).

Givosiran is an FDA-approved therapeutic siRNA based on GalNAc-siRNA technology. As previously discussed, givosiran is indicated for AHP caused by a disorder of hepatic ALAS1 and acute intermittent porphyria (AIP), which can result in the accumulation of several components, including toxic metabolites such as porphyrin precursors, ALA and PBG, leading to multiorgan and multisystem injury, including nervous system injury, along with conditions such as chronic kidney disease, hepatocellular carcinoma and hypertension (Lazareth et al., 2021). Iron overload and vein thrombosis are the predominant limitations of intravenous hemin application to suppress ALAS1 induction and cure palindromic attacks. After incorporation into RISC, givosiran then adopts the RNAi mechanism to silence the mRNA of hepatic ALAS1, thus thwarting the synthesis of ALAS1 integrin (Bissell and Wang, 2015; Sawicki et al., 2015; Agarwal et al., 2020; de Paula Brandão et al., 2020; Lazareth et al., 2021). During phase three testing of the ENVISION trial, the givosiran-injected group exhibited a lower rate of annual attacks, lower urinary ALA levels, and less pain than the placebo. Nonetheless, higher serum transaminase levels and creatinine levels were observed, attributed to glomerular filtration changes (Balwani et al., 2020; Bonkovsky et al., 2020; Ventura et al., 2021). Overall, givosiran is still paving the way for the clinical application of GalNAc-siRNA conjugates and represents an excellent AIP oligonucleotide therapeutic option that can promote siRNA development.

Inclisiran is another GalNAc-siRNA conjugate approved by the FDA that can bind to hepatically expressed PCSK9 proteins, representing ideal targets that affect systemic and regional lipid metabolism and plasma cholesterol level regulation (Leiter et al., 2019; German and Shapiro, 2020; Kam et al., 2020; Scicchitano et al., 2021). Inclisiran has been documented to be effective in lowering LDL-C in cases of high cholesterol levels. Importantly, drug elimination 24 h after injection and liver uptake selectivity and efficiency suggest inclisiran to be a promising drug (Tomlinson et al., 2021). Research has shown that inclisiran and bempedoic acid can resolve limitations associated with pH treatment (Aguilar-Salinas et al., 2021). Moreover, it has been shown that inclisiran can decrease LDL-C levels; the published ORION trials demonstrated that combining inclisiran and the maximum tolerated dose of statins led to a 150% decline in LDL-C (Stoekenbroek et al., 2018; Gallego-Colon et al., 2020; Bardolia et al., 2021; Hardy et al., 2021).

Lumasiran targeting glycolate oxidase is a promising therapeutic agent shown to effectively lower oxalate levels in the liver, resulting in less deposition of calcium oxalate crystals in the kidneys and lower probability of kidney failure, systemic oxalosis and other systemic organ failure due to metabolic dysfunction (Perazella and Herlitz, 2021; Scott and Keam, 2021; Shah et al., 2021). Glyoxylic acid is obtained by glycolic acid conversion catalyzed by glycolate oxidase and is inhibited by limiting glyoxylate availability, leading to increased glycolate levels (Dutta et al., 2016; Liebow et al., 2017; Frishberg et al., 2021). A phase 1/2 randomized placebo-controlled study showed that lumasiran was biologically safe and effectively reduced urinary oxalate excretion in all cases with PH1 during the trial (Frishberg et al., 2021). In a phase three double-blind trial where urinary oxalate excretion patients with PH1 were studied, lumasiran effectively reduced progression to kidney failure (Garrelfs et al., 2020; Garrelfs et al., 2021; Frishberg et al., 2022).

Vutrisiran is a transthyretin-directed siRNA therapeutic for the treatment of amyloid transthyretin-mediated (ATTR) amyloidosis, including hATTR amyloidosis and wild-type ATTR (wtATTR) amyloidosis (Keam, 2022). Vutrisiran reduces serum TTR levels by reducing synthesis of variant and wild-type TTR which primarily synthesis in liver. Via ESC design of GalNAc-siRNA conjugates, Vutrisiran is able to bind ASGPR effectively, allowing for once every 3 months subcutaneous injection with better metabolic stability and improved potency in a phase three study (Soprano et al., 1985; Holmgren et al., 1991; Habtemariam et al., 2021; Alnylam Pharmaceuticals Inc, 2022).

#### 3.3.2 Clinical and preclinical advances in GalNAc-siRNA therapeutics

As the representative of GalNAc conjugates clinical usage, Fitusiran, developed by Alynlam and Sanofi Genzyme, is a synthetic siRNA targeting liver antithrombin to increase thrombin generation developed for the treatment of coagulation factor VIII deficiency (hemophilia A) and coagulation factor IX deficiency (hemophilia B) and is ongoing. In a phase 1 inhibitor cohort, monthly fitusiran lowered antithrombin levels from baseline, resulting in thrombin generation improvements, suggesting that monthly subcutaneous injections of fitusiran may lead to bleeding episode reduction and improved quality of life in participants with hemophilia A or B treated with inhibitors (Pasi et al., 2021).

The phase three ATLAS trial design consists of major branches including 1) ATLAS-A/B (NCT03417245), assessing fitusiran or on-demand factor replacement therapy, and 2) ATLAS-INH (NCT03417102), assessing fitusiran or on-demand bypassing agent therapy (Zhang et al., 2021a). A phase three results for 1) ATLAS-A/B (Genzyme, 2021) and 2) ATLAS-INH (Genzyme, 2020) are completed, with outcomes summarized. In ATLAS-A/B and ATLAS-INH, the observed annualized bleeding rate (ABR) for treated bleeds during the efficacy period and the treatment period and the onset period of the fituriran 80 mg prophylaxis group are significantly reduced compared to the on-demand group. The same tendency also appears in the observed annualized spontaneous bleeding rate and annualized joint bleeding rate for treated bleeds during the efficacy period, representing less spontaneous bleeding events appearing after subcutaneous administration of 80 mg (mg) of fitusiran as prophylaxis once monthly compared to the on-demand or bypassing agents (BPA) on-demand group.

Additionally, preclinical undergone experiments with published papers are listed, with comparisons on years, models, animals, etc. (Willoughby et al., 2018; Brown et al., 2020; Ayyar et al., 2021; Iwakawa and Tomari, 2021; Yang et al., 2021) (Supplementary Table S3).

### 3.4 Challenges and limitations

Although GalNAc conjugates possess better stability, other crucial factors, such as endosomal escape, hepatotoxicity from off-target effects, acidic subcellular compartments, and extensive clearance, affect GalNAc efficiency and sequence barriers (Ayyar et al., 2021; Fairman et al., 2021; Fattal and Fay, 2021; Fumoto et al., 2021; Liu et al., 2021; Nanavati et al., 2021; Schlich et al., 2021).

#### 3.4.1 Endosomal escape

When GalNAc conjugates are functional *in vivo*, only a few free siRNAs can escape into the hepatocyte cytoplasm and cross the endosomal lipid bilayer membrane, allowing siRNA to transactivate reactive RNA-binding proteins and resulting in a rapid, robust and sustained RNAi response by loading onto the host cell Ago (Springer and Dowdy, 2018). Endosomal escape is considered the rate-limiting step, preventing GalNAc conjugates from exerting their effects. Cell-penetrating peptides (CPPs) are short peptides that cross cellular membranes, facilitating endosomal escape (Oyama et al., 2021). Lonn et al. (2016) hypothesized that the insertion of a lipid bilayer with a hydrophobic patch into PTD/CPP-EED domains resulted in localized membrane destabilization that enhanced endosomal escape into the cytoplasm. CPPs exhibit heterogeneous rates of toxicity, penetration, and membrane leakage (Lonn et al., 2016; Benizri et al., 2019).

#### 3.4.2 Hepatotoxicity from off-target effects

Janas et al. (2018b) hold a point of view that during supratherapeutic exposures, instead of chemical modifications or the perturbation of RNAi pathways, the observed rodent hepatotoxicity can be largely attributed to RNAi-mediated off-target effects. Theoretically, full-length pairing targets mRNA distinctively, while in the siRNA guide strand at positions 2–8 of the seed region (g2-g8), there are complementary sites that bind to the 3′ untranslated region (3′-UTR) of mRNAs: thereby, unexpected off-target effects occur through the combination of the two, which is capable of leading to enormous dysregulation of transcription through a manner similar to miRNA (Schlegel et al., 2021; Varley and Desaulniers, 2021). Hepatotoxicity not only results from off-target effects caused by genetic changes, but is also related to intracellular oligonucleotide accumulation and chemical reactivity with metabolites. Moreover, although hepatotoxicity can be associated with disturbances during the RNAi process, it is mainly attributed to off-target effects (Janas et al., 2018b).

To eliminate hepatotoxicity, several solutions have been found. Janas et al. provide compelling evidence that off-target effects play key roles in hepatotoxicity during GalNAc treatment in rats (Janas et al., 2018a; Janas et al., 2018b). It was found that during seed-mediated binding, thermal destabilization is a feasible option to reduce *in vivo* siRNA off-target effects, thereby reducing hepatotoxicity. It is possible to selectively infuse a single thermally destabilizing GNA nucleotide into the seed region of the antisense strand to reduce seed-mediated off-target binding, thereby improving the safety of GalNAc-conjugated siRNA in rats (Janas et al., 2018b). Xu et al. (2004) suggested that among the factors that determine siRNA efficiency, siRNA molecular structural characteristics, such as single or duplex-stranded nature, might be more significant than cellular persistence. In this regard, it has been shown that the duplex siRNA yielded better efficiency of RISC reconstitution versus single-stranded siRNA, which paved the way for further investigations due to concerns about the use of structural features to improve transfection efficiency (Xu et al., 2004).

#### 3.4.3 Acidic subcellular compartment

It is shown that the results of GalNAc-siRNA conjugate loading into RISC match the corresponding model of GalNAc-siRNA conjugate liberation from the acidic subcellular compartment (Fairman et al., 2021; Schlich et al., 2021). Since less stable siRNA designs degrade faster in acidic subcellular compartments, such as lysosomes, they are unable to support continuous RISC loading over time. Brown et al. (2020) found that the predominant driver for the extended duration of activity is increasing the half-life of chemically stabilized siRNA in acidic subcellular compartments. They also proposed that a slow release of stabilized siRNA from acidic subcellular compartments enables continuous loading of RISC and prolonged target silencing. Nonetheless, enhancing siRNA can counter aggressive degradation of the intracellular compartments by nucleases, leading to better knockdown efficiency and a prolonged effective period.

#### 3.4.4 Extensive enzymatic and/or systemic clearance

Given the wide range of nuclease degradation, sufficient RNA packaging is crucial to ensure that modified RNA can properly access receptors. Additionally, inadequate oligonucleotides exhibit quick renal filtration and elimination, leading to less intracellular residues and a lower half-life, significantly limiting treatment efficacy (Kovacevic et al., 2018; Ayyar et al., 2021; Fairman et al., 2021; Nanavati et al., 2021; Yan et al., 2022).

#### 3.4.5 Sequence barriers

For many siRNA targets, the use of animal models to predict clinical results is prohibited because of the differential sequences between preclinical species. Therefore, new preclinical approaches are necessary in the development of oligonucleotide therapeutics to allow evaluation of target cell delivery as well as RNA silencing efficacy in species-relevant systems (Li, 2010). Primary human hepatocytes, which are considered the “gold standard” *in vitro* experimental model for the evaluation of small molecule drug metabolism, drug-drug interactions, and toxicity, are recommended for siRNA with hepato-cellular targets based on the limitations of the animal models.^110^ Primary human hepatocytes enable good effects with respect to potency and duration for the evaluation of GalNAc-siRNA efficacy due to the significant species (human) and organ (liver) relevance. Yang et al. collected findings that demonstrate the potential utility of prolonged cultured human hepatocytes (PCHH) as a preclinical tool for the translation of hepatocyte-targeted siRNA, with advantages such as long culture duration, lack of cell division and prolonged expression of hepatic transcripts in a species, organ and pharmacologically relevant system. In their POC study, robust, durable, HPRT1 mRNA knockdown was demonstrated with a GalNAc-conjugated, stability-enhanced siRNA molecule, indicating that all biochemical systems required to facilitate siRNA delivery and activity are intact and functional in the cryopreserved 999 Elite Human Hepatocytes used in the establishment of PCHH. This *in vitro* model may serve as a valid alternative for siRNA prioritization and selection to reduce overall animal usage in addition to the provision of human hepatocyte-specific results, which may not be readily obtainable from *in vivo* animal models (Yang et al., 2021).

## 4 Other delivery systems

However, it was found that after years of development, available siRNA drugs still cannot solve problems associated with endosomal escape barriers, off-target effects and extensive enzymatic and/or systemic clearance. The targeting precision needs to be further improved (Xu et al., 2004; Lonn et al., 2016; Janas et al., 2018b; Kovacevic et al., 2018; Zhang et al., 2018; Benizri et al., 2019; Jahns et al., 2021; Schlegel et al., 2021; Yan et al., 2022). Accordingly, further research and development efforts are needed to apply this technology in the clinic.

### 4.1 LNPs loaded siRNA

The LNP delivery system, also known as the second-generation delivery system of RNAi drugs, is mainly composed of four parts: polyethylene glycol-lipid conjugates (such as PEG-DMG), ionizable amino lipids (such as DLin-MC3-DMA), distearyl phosphatidylcholine (DSPC) and cholesterol (Aldosari et al., 2021; Lokugamage et al., 2021). PEG-lipid on the outside helps protect the contents, while the cationic lipids inside can electrostatically adsorb genetic materials. The oligonucleotide drugs are protected by encapsulation, forming a cationic lipid package (Eygeris et al., 2021; Li et al., 2022; Paunovska et al., 2022; Yan et al., 2022). Cholesterol near the cationic lipid package can help stabilize the structure of nanoparticles and assist endocytosis induced by LDL (Gupta et al., 2021b; Zhang et al., 2021b; Chaudhary et al., 2021; Huang et al., 2021). Overall, the LNP delivery system is currently one of the most effective siRNA delivery methods and is mainly used for intravenous administration (Carrasco et al., 2021; Everton et al., 2021). During the transport of LNP-encapsulated siRNA into the body, LNP first fuses with the lipid bilayer of the cell membrane and then releases siRNA into the cell, allowing systemic siRNA administration (Table 2) (Wang et al., 2021a; Yan et al., 2022).

**TABLE 2:** Comparison of GalNAc- siRNA and LNP loaded siRNA.

	GalNAc-siRNA	LNPs loaded siRNA (Aldosari et al., 2021; Zhang et al., 2021a; Lokugamage et al., 2021)
Benefits & Challenges	-can be injected subcutaneously, with only small chances of plasma siRNA degradation, rapid absorption, high uptake, and long half-life, (Springer and Dowdy, 2018)-endosomal escape, hepatotoxicity from off-target effects, acidic subcellular compartments, and extensive clearance, affect GalNAc efficiency and sequence barriers (Ayyar et al., 2021; Fairman et al., 2021; Fattal and Fay, 2021; Fumoto et al., 2021; Liu et al., 2021; Nanavati et al., 2021; Schlich et al., 2021)	-avoid siRNA degradation of and the stimulation of the immune system by siRNA-lack efficient nuclear penetration and sustainable transgene expression (limitations such as poor biodistribution and possible toxic discharge), immunogenic and pose safety concerns of liposome molecules and limited efficacy and biosafety since unconjugated liposomes cannot achieve targeted delivery (Ickenstein and Garidel, 2019; Hu et al., 2020; Blakney et al., 2021; Hassett et al., 2021; Maestro et al., 2021)
Composition	Specific arrangement of nucleoside, *p*O/PS linkage and monovalent GalNAc (Matsuda et al., 2015)	polyethylene glycol-lipid conjugates (PEG-DMG), ionizable amino lipids (DLin-MC3-DMA), distearyl phosphatidylcholine (DSPC) and cholesterol
Attachment	Triantennary GalNAc ligand	PEGylated surface
Target	ASGPR on hepatocyte	Liver and Triple-negative breast cancer (TNBC) (Wang et al., 2021b)
RNAi activity (Brown et al., 2020)	slower	faster
RNAi action time (Brown et al., 2020)	longer	shorter
Approved drugs	Inclisiran, Lumasiran, Givosiran, Vutrisiran etc.	Patisiran (Weng et al., 2019), etc.

### 4.2 ESC and advanced ESC loaded by LNPs

GalNAc-siRNA conjugates are transmitted by ASGPR-mediated endocytosis and accumulate in acidic cells. It is mentioned that high metabolic stability is the most vital factor for GalNAc-siRNA to achieve optimal activity *in vivo* (Foster et al., 2018). Moreover, after subcutaneous injection of a certain dose of ESC and advanced ESC conjugate, significantly different pharmacodynamic characteristics were observed; the stability of advanced ESC was higher than that of ESC. Although studies have shown that ESC conjugates yield a stronger effect at maximum knockout after a threefold increase in the dose, advanced ESC conjugates exhibit more enduring activity, which proves that greater stability of the chemical properties of GalNAc-siRNA enables longer duration of action (Springer and Dowdy, 2018). Furthermore, a study that directly compared the effects of GalNAc coupling and LNP delivery on the siRNAs of traditional and advanced ESC targeting mouse factor 7 indicated that during LNP delivery, ESC and advanced ESC with similar intrinsic efficacy exhibited similar behaviors, which indicates that LNP delivery eliminated the differences in time and efficiency between traditional and advanced ESC. Besides, the exposure levels of total siRNA in the liver after subcutaneous injection of ESC and advanced ESC were lower than those of ESC and advanced ESC delivered by LNPs. It is worth noting that the delivery mode of ESC and advanced ESC loaded with LNPs occurred *via* intravenous injection, substantiating that the delivery mode can significantly affect the activity and duration of GalNAc-siRNA (Brown et al., 2020; Kulkarni et al., 2021; Naito et al., 2021; Salim and Desaulniers, 2021).

## 5 Conclusion

Many chronic or acute serious diseases are associated with the liver, such as liver cancer, hepatitis, hepatic hemangioma, etc., which remain among the leading causes that seriously affect the quality of human life and even lead to human death. The immense potential of GalNAc siRNA conjugates in the treatment of liver-related diseases involving gene expression provides a new strategy for this purpose. Through years of improvement, siRNA therapeutics have achieved superior advances in delivery accuracy and targeting precision, and both LNP siRNA and GalNAc siRNA delivery systems have enabled siRNA systemic delivery. However, GalNAc conjugate-based siRNA capable of direct targeted delivery offers the advantages of being safer and more efficient than the second-generation delivery system LNP siRNA. Additionally, the results of intravenous injection of GalNAc-siRNA highlighted the durability of RNAi, while subcutaneous injection increased target gene knockout and prolonged the duration of RNAi activity. Moreover, studies have shown that chemically stable siRNA persists in highly acidic subcellular compartments after administration, with chemical stability contributing to prolonging the activity of RNAi. The progress in siRNA design and chemical modification is the crucial step in improving the stability of siRNA metabolism when designing oligonucleotides with continuously increased efficacy and enhancing the half-life of the GalNAc conjugates. To date, with several approved drugs and ongoing clinical and preclinical trials, it is promising that with more contributions taken into account, more delicate designs and modifications can lead GalNAc conjugates to not only longer duration but also higher efficiency, reduced toxicity and tighter attachment. Challenges such as endosomal escape barriers, off-target effects, and a lack of safety profile remain hindrances to actualizing the full opportunity and potential of oligonucleotide drugs. Thankfully, massive research is underway to investigate these obstacles, including the development of hepatic and extrahepatic delivery platforms, paving the way for further design and development. It is believed that state-of-the-art siRNA technology will surely be a boon to clinical medicine.
