# Supplementary material for: The therapeutic prospects of N-acetylgalactosamine-siRNA conjugates
Source: Front Pharmacol. 2022 Dec 14;13:1090237. doi: 10.3389/fphar.2022.1090237 (PMC9794871; doi:10.3389/fphar.2022.1090237)
Supplement: Supplementary file 1 [file DataSheet1.docx]

**Table S1. GalNAc-siRNA preclinical development activities.**

| **Code Name** | **Product Last Updated** | **Molecular Mechanism** | **Product Category** | **Condition** | **Company** |
| --- | --- | --- | --- | --- | --- |
| OLX-703A | Oct 29, 2021 | Drugs Targeting Hepatitis B Virus (HBV) Proteins | RNA siRNAs | Hepatitis B (HBV) | OliX Pharmaceuticals |
| GNV-705 | Aug 24, 2021 | n/a | RNA siRNAs | Acromegaly | Genevant Sciences |
| GNV-069 | Aug 21, 2021 | n/a | RNA siRNAs | Fibrosis, hepatic | Genevant Sciences |
| OLX-702-A | Apr 13, 2022 | n/a | RNA siRNAs | Non-alcoholic steatohepatitis (NASH) | OliX Pharmaceuticals |
| ALG-125755 | Mar 26, 2022 | HBsAg Expression Inhibitors | RNA siRNAs | Hepatitis B, chronic | Aligos Therapeutics |
| DCR-LIV2 | Feb 19, 2022 | n/a | RNA RNA Interference | Non-alcoholic steatohepatitis (NASH) | Boehringer Ingelheim |
| MT-001 | Feb 19, 2022 | DNAJC15 (MCJ) Expression Inhibitors | RNA siRNAs | Non-alcoholic steatohepatitis (NASH) | Mitotherapeutix |
| ALN-AC3 | Feb 27, 2017 | n/a | Oligonucleotides siRNAs | Hypertriglyceridemia | Alnylam Pharmaceuticals |

(n/a: not applicable).

**Table S2. GalNAc-siRNA clinical development activities.**

| **Code Name** | **Product Last Updated** | **Highest Phase** | **Molecular Mechanism** | **Product Category** | **Condition** | **Company** |
| --- | --- | --- | --- | --- | --- | --- |
| pozelimab/cemdisiran  (Generic Name) | Nov 17, 2021 | Phase III | Anti-C5 (Complement 5) C5 Expression Inhibitors | RNA Fixed-Dose Combinations Human Monoclonal Antibodies siRNAs | Myasthenia gravis Paroxysmal nocturnal hemoglobinuria | Alnylam Pharmaceuticals (Originator)  Regeneron (Originator) |
| AKCEA-APO(a)-LRx IONIS-APO(a)-LRx ISIS-681257 ISIS-APO(a)-LRx LSO9H7UZ90 TQJ-230 | May 3, 2022 | Phase III | APOA Expression Inhibitors | Antisense Therapy Oligonucleotides | Atherosclerosis Cardiovascular Disorders Hyperlipidemia Lipid metabolism disorders | Akcea Therapeutics (Ionis Pharmaceuticals)  Ionis Pharmaceuticals (Originator)  Isis Pharmaceuticals (Ionis Pharmaceuticals)  Novartis |
| AD-62643  ALN-62643  ALN-CC5  S66Z65E10T | May 18, 2022 | Phase III | C5 Expression Inhibitors | RNA  siRNAs | Complement-mediated disease  Hemolytic uremic syndrome  Hemolytic uremic syndrome, atypical  IgA nephropathy  Membranous nephropathy  Myasthenia gravis  Paroxysmal nocturnal hemoglobinuria | Alnylam Pharmaceuticals (Originator)  Regeneron |
| AD-57213  ALN-AT3  ALN-AT3SC  GalNAc-AT siRNA  SV9W47ZLE1 | Mar 5, 2022 | Phase III | SERPINC1 Expression Inhibitors | RNA  siRNAs | Hemophilia B  Hemophilia A | Alnylam Pharmaceuticals (Originator)  Genzyme (Sanofi Genzyme)  Sanofi  Sanofi Genzyme |
| AB-729  ARB-270729 | Dec 27, 2021 | Phase II | HBsAg Expression Inhibitors | DNA  siRNAs | Hepatitis B, chronic | Arbutus Biopharma (Originator)  Vaccitech Ltd. (Vaccitech plc) |
| BAY-2976217  FXI-LICA  ION-957943  IONIS-FXI-LRx | Mar 24, 2022 | Phase II | F11 (FXI) Expression Inhibitors | Antisense Therapy  DNA | Thrombosis | Bayer  Ionis Pharmaceuticals (Originator) |
| CiVi-007  LNA-PCSK-9  LNA-PCSK9-GalNac | Jun 5, 2020 | Phase II | PCSK9 Expression Inhibitors | Antisense Therapy  Locked Nucleic Acids | Cardiovascular Disorders  Hypercholesterolemia | CiVi Biopharma  Roche (Originator) |
| ALN-AAT02 | Jan 15, 2019 | Phase I/II | SERPINA1 (Z-allele Mutant) Expression Inhibitors | RNA  siRNAs | Alpha-1 antitrypsin deficiency | Alnylam Pharmaceuticals (Originator) |
| SLN-124 | Mar 26, 2022 | Phase I/II | TMPRSS6 Expression Inhibitors | RNA  siRNAs | Hemochromatosis  Iron overload  Myelodysplasia  Polycythemia vera  Thalassemia, beta | Silence Therapeutics (Originator) |
| ALN-XDH | Apr 20, 2022 | Phase I/II | Xanthine Dehydrogenase/Oxidase (XDH; XOR) Inhibitors | RNA  siRNAs | Gout | Alnylam Pharmaceuticals (Originator) |
| ALN-AAT  GalNAc-AAT | Oct 4, 2018 | Phase I/II | SERPINA1 (Z-allele Mutant) Expression Inhibitors | RNA  siRNAs | Alpha-1 antitrypsin deficiency | Alnylam Pharmaceuticals (Originator) |
| DCR-AUD | Feb 8, 2022 | Phase I | ALDH2 Expression Inhibitos | RNA  RNA Interference | Alcoholism | Dicerna (Originator) |
| LY-3819469 | Feb 19, 2022 | Phase I | LPA Expression Inhibitors | RNA  siRNAs | Metabolic Diseases | Dicerna (Originator)  Lilly |
| SLN-360 | Mar 26, 2022 | Phase I | APOA Expression Inhibitors | RNA  siRNAs | Lipid metabolism disorders  Lipoprotein disorders | Silence Therapeutics (Originator) |

**Table S3. Preclinical advances in GalNAc-siRNA therapeutics**

| **Year** | 2021 | | **Model** | | Liver mPBPK-PD Model, Cellular mPBPK-PD Model | **Animals** | | mice, rats, cynomolgus monkeys, humans | | |
| --- | --- | --- | --- | --- | --- | --- | --- | --- | --- | --- |
| **Duration** | n/a | | **Cell Lines** | | primary cultured hepatocytes from mice | **Modalities** | | fitusiran (ALN-AT3) | **Citation** | 1 |
| **Breakthroughs** | | 1) development of a minimal PBPK-PD (mPBPK-PD) model to comprehensively characterize the whole-body–to-intracellular PK-PD actions of GalNAc-siRNA,  2) physiological scaling of the platform model across three preclinical species (mouse, rat, and monkey),  3) characterization of clinical response in a first-in-human study for a specific GalNAc-siRNA,  4) demonstration of the utility of translational systems modeling to quantitate the PK-PD properties of this novel therapeutic platform. | | | | | | | | |
| **Boundedness** | | 1) determination of whether interspecies differences in lymphatic physiology could explain the observed trends in *k_a_* after subcutaneous dosing requires further exploration,  2) reported subcutaneous bioavailability estimates are underestimated as a result of an early, transient saturation of ASGPR after higher intravenous doses. | | | | | | | | |
| **Year** | 2020 | **Model** | | | n/a | **Animals** | | Female C57BL/6 mice ~6–8 weeks of age | | |
| **Duration** | 77 days | **Cell Lines** | | | Primary mouse hepatocytes | **Modalities** | | ESC, advanced ESC | **Citation** | 2 |
| **Breakthroughs** | | 1) More stable metabolism and slower release of the siRNA correlate well with duration of activity, slowing degradation in the acidic compartments, and providing constant RISC loading.  2) With higher total hepatic siRNA levels, advanced ESC performs better than ESC in terms of duration of target knockdown. | | | | | | | | |
| **Boundedness** | | 1) Potential benefits in duration and target knockdown can be lost if the chemical modification is misplaced.  2) Inability to rule out other cellular sites of siRNA accumulation due to the limited half-life in mice.  3) Osmotic agents, cell penetrating and fusogenic peptides, melittin, dfTAT and photochemical stimulation help increase siRNA release but are impractical due to toxicity. | | | | | | | | |
| **Year** | 2020 | **Model** | | | n/a | **Animals** | | n/a | | |
| **Duration** | 41 days | **Cell Lines** | | | Cryopreserved 999 Elite human hepatocytes | **Modalities** | | GalNAc-siRNA | **Citation** | 3 |
| **Breakthroughs** | | GalNAc-conjugated siRNA was effectively endocytosed by the hepatocytes and delivered to the nucleus to enable robust and persistent silencing of HPRT1 mRNA as proﬁled over a comprehensive time course, with the following conclusions: 1) lack of toxicity of the GalNac-conjugated siRNA, 2) siRNA-treated hepatocytes showing similar WST metabolism activity with respect to the untreated control throughout culture duration, 3) apparent speciﬁcity of the GalNac-siRNA silencing HPRT1, and not for the nontarget genes ALB, TF and TTR, showing noninterference with normal hepatic gene expression, 4) primary cultured human hepatocytes may be used for the evaluation of factors regulating ASGR-1 expression, which may aid the design and implementation of GalNac-siRNA therapeutics. | | | | | | | | |
| **Boundedness** | | No cell division and without species cross-reactivity concerns. | | | | | | | | |
| **Year** | 2017 | **Model** | | Model generated by using an *in vitro* silencing dataset of 1,890 duplexes with varying 2’-F and 2’-OMe composition across 5 targets and 15 target sites, describing the impact of 2’-F relative to 2’-OMe at each position in the antisense and sense strands. | | | **Animals** | 8-week-old C57BL/6 female mice, nonhuman primate | | |
| **Duration** | ≥100 days | **Cell Lines** | | Cryopreserved primary mouse hepatocytes | | | **Modalities** | DV18, DV22 | **Citation** | 4 |
| **Breakthroughs** | | 1) Minor changes in chemical modification can significantly affect metabolic stability, thus affecting the *in vivo* performance of the GalNAc conjugates.  2) There is a certain degree of chemical interdependence between the complementary strands of the siRNA duplex for optimal silencing activity.  3) Both designs, DV 18 and DV 22, were considered as low 2'-F content that achieved improved *in vivo* efficacy and duration, proving that enhanced modification can provide both better performance and lower cost.  4) Increased efficacy in both mouse and nonhuman primate. | | | | | | | | |
| **Boundedness** | | Not yet the best modification since substantially larger datasets are required and the effects of neighboring modifications and overall modification of siRNA patterns are not under consideration. | | | | | | | | |
| **Year** | 2017 | **Model** | | | EtOH-Diet Mouse Model, PB-Induced Liver Injury Model | **Animals** | | C57BL/6 female mice, aged 6–8 weeks, randomly assigned *Asgr2*^-/-^ animals of 6 to 12 weeks of age in each group | | |
| **Duration** | 11 days | **Cell Lines** | | | n/a | **Modalities** | | GalNAc-siTTR | **Citation** | 5 |
| **Breakthroughs** | | 1) Potential application of GalNAc-siRNA even in disease settings that may include lower receptor levels.  2) Potent GalNAc-conjugated siRNAs should be functional in the context of AAT-PiZZ fibrotic livers, the altered-architecture liver.  3) Potential application of GalNAc conjugates even in the context of lower receptor levels in some diseases. | | | | | | | | |
| **Boundedness** | | n/a | | | | | | | | |

(n/a: not applicable).

**References**

1. Ayyar, V. S.; Song, D.; Zheng, S.; et al. Minimal Physiologically Based Pharmacokinetic-Pharmacodynamic (mPBPK-PD) Model of N-Acetylgalactosamine–Conjugated Small Interfering RNA Disposition and Gene Silencing in Preclinical Species and Humans. J. Pharmacol. Exp. Ther. 2021, 379, 134–146.

2. Brown, C. R.; Gupta, S.; Qin, J.; et al. Investigating the Pharmacodynamic Durability of GalNAc-siRNA Conjugates. Nucleic Acids Res. 2020, 48, 11827–11844.

3. Yang, Q.; Humphreys, S. C.; Lade, J. M.; et al. Prolonged Cultured Human Hepatocytes as an In Vitro Experimental System for the Evaluation of Potency and Duration of Activity of RNA Therapeutics: Demonstration of Prolonged Duration of Gene Silencing Effects of a GalNAc-Conjugated Human Hypoxanthine Phosphoribosyl Transferase (HPRT1) siRNA. Biochem. Pharmacol. 2021, 189, 114374.

4. Iwakawa, H.-O.; Tomari, Y. Life of RISC: Formation, Action, and Degradation of RNA-Induced Silencing Complex. Mol. Cell 2021, 82, 30–43.

5. Willoughby, J. L.; Chan, A.; Sehgal, A.; et al. Evaluation of GalNAc-siRNA Conjugate Activity in Pre-Clinical Animal Models with Reduced Asialoglycoprotein Receptor Expression. Mol. Ther. 2018, 26, 105–114.
